# The Quest for Oral PROTAC drugs: Evaluating the Weaknesses of the Screening Pipeline

**DOI:** 10.1021/acsmedchemlett.3c00231

**Published:** 2023-07-03

**Authors:** Giulia Apprato, Giuseppe Ermondi, Giulia Caron

**Affiliations:** Molecular Biotechnology and Health Sciences Department, University of Torino, Piazza Nizza 44, 10124 Torino, Italy

**Keywords:** Permeability, Physicochemical descriptors, Preclinical pipeline, PROTAC, Solubility

## Abstract

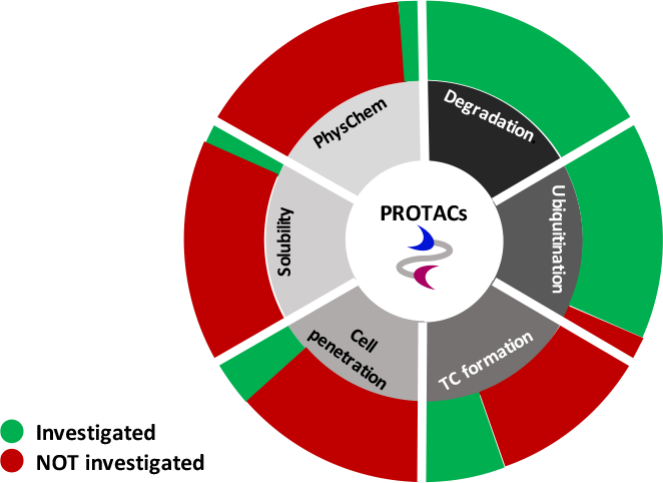

A targeted bibliographic search exposed the deficiencies within existing PROTAC preclinical pipelines, including missing, poor-quality data and technical limitations in the experimental assays. Several recommendations are proposed to improve the efficiency of preclinical platforms for PROTACs.

PROTACs (Proteolytic Targeting Chimeras), hereafter often addressed as degraders, are heterobifunctional molecules capable of inducing E3 ligase-mediated ubiquitination and subsequent degradation of a target protein (protein of interest or POI). Their unconventional catalytic mode of action and the associated advantages made PROTACs a new therapeutic modality, awakening huge interest in drug discovery.^1,2^ The potential of PROTACs to address undruggable targets such as proteins with shallow surfaces, often involved in protein–protein interaction (PPI), and even scaffold proteins, other than the ability to target resistant cancer forms,^3,4^ piqued the interest of both pharma/biotech industries and academia.^5,6^ Remarkably, PROTACs possess large and flexible structures, which introduce notable challenges in concurrently optimizing solubility and cellular permeability. Specifically, the pursuit of increased permeability through heightened lipophilicity can potentially result in diminished solubility and metabolic stability.^7,8^

A recent bibliometric analysis pointed out that in the last 20 years more than 800 PROTAC-related papers have been published, involving the contribution of 3886 authors worldwide.^9^ As a result, a significant quantity of chemical matter has been generated (of note, the selection of building blocks, and synthetic strategies are beyond the aim of this paper). However, just a limited number of compounds with potential for development emerged, leading to a scarcity of candidates entering clinical trials. In our opinion, the lack of a well-defined experimental pipeline involving default protocols deeply hinders the rational design of new candidates. The vast heterogeneity of information is particularly evident when analyzing PROTAC-DB, the most comprehensive repository of PROTAC-related structures and experimental data.^10^

## The PROTAC Action and the Related Experimental Pipeline

**Figure 1:**
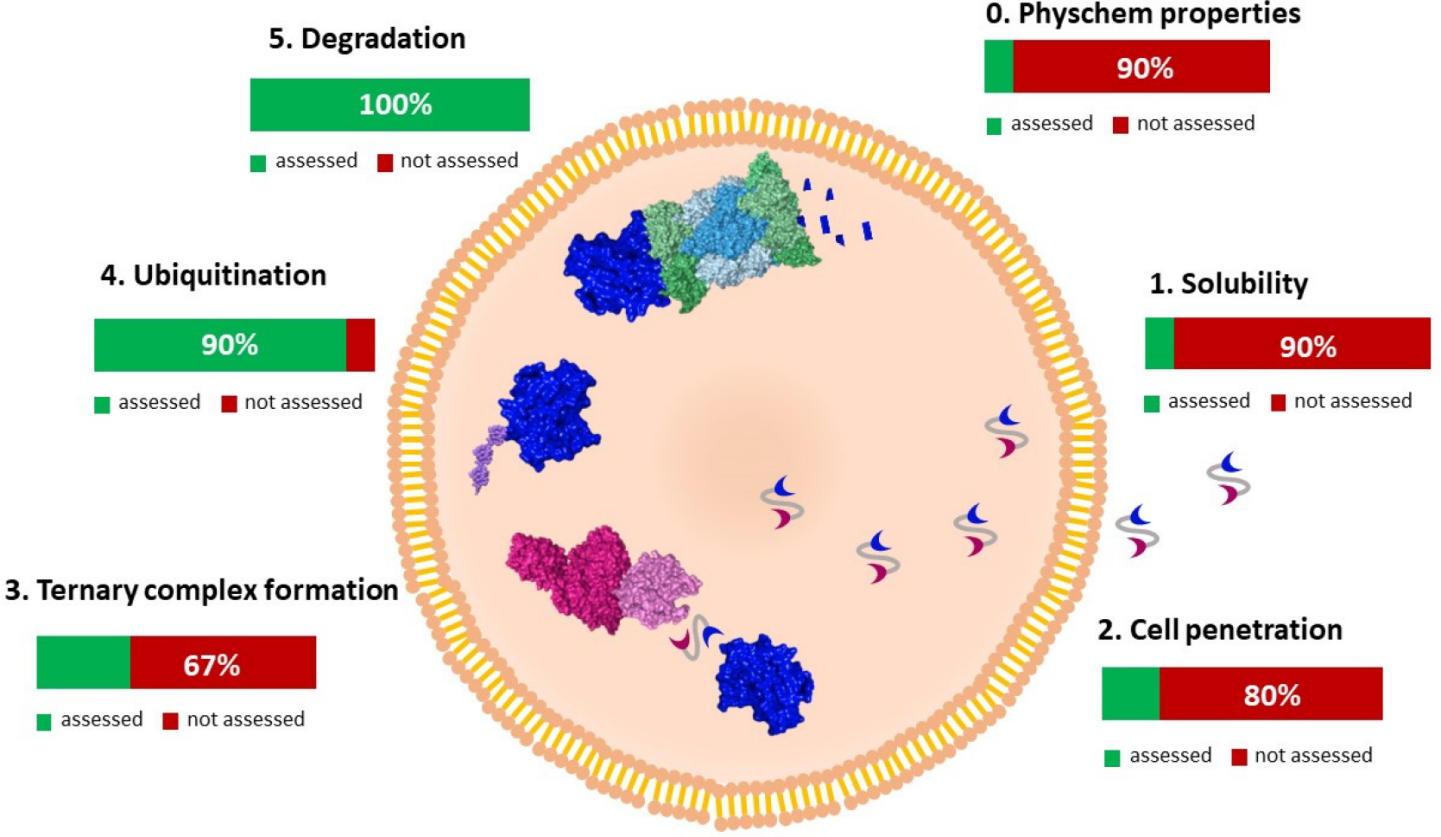
Schematic of PROTAC action: main steps and their relative frequency as calculated from data reported in the retrieved papers.

In general terms, the mechanism of action of a PROTAC drug involves its cellular entry (in turn related to solubility, permeability and physicochemical properties), the formation of the ternary complex, the ubiquitination of the POI, and ultimately the POI degradation through the proteasome pathway (steps 0–5 in Figure 1).

Since all steps in Figure 1 are mandatory to guarantee the degrader efficacy, PROTAC platforms are expected to collect experimental data related to all of them. Therefore, at least in principle, the experimental preclinical screening pipeline should measure a pool of physicochemical (e.g., ionization and lipophilicity) and in vitro ADME (i.e., solubility and permeability) descriptors related to cell penetration (step 0–2 in Figure 1), verify the ternary complex formation (step 3), confirm the involvement of the ubiquitin-proteasome system (step 4), and assess the POI degradation (step 5). Missing one or more of these steps risks jeopardizing the success of the program.

In an effort to identify the gaps in PROTAC experimental pipelines and offer guidelines to enhance preclinical screening platforms, we conducted a bibliographic research (details in the Supporting Information) focusing on selected up to date PROTAC-related literature, i.e., papers published in *Journal of Medicinal Chemistry* between January 1, 2021 and March 15, 2023. Overall, we retrieved 112 papers (temporal distribution in Figure S1A; data reported in Tables S1 and S2). Although an upward trend is not visible, one-half of the articles were published last year, and one-third in the last 6 months. These publications include 61 articles, 48 perspectives, 2 editorials, and 1 drug annotation. A manually curated analysis allowed us to identify 37 articles reporting PROTAC-related data (target distribution in Figure S1B,C). We also applied the same strategy to *ACS Medicinal Chemistry Letters*. We retrieved 59 publications, but only 10 of them could be considered pertinent (details in the Supporting Information).

## Data Produced by Experimental Pipelines

Experimental evidence related to steps 0–5 were first extracted from the 37 *J. Med. Chem.* papers; their relative frequency is reported in Figure 1. It must be noticed that steps 0–2 are poorly considered: most papers do not include any information concerning solubility and cell permeability, 10% reported about experimental/predicted molecular properties, just 20% measured PROTAC cellular penetration, and 10% investigated solubility, with 3 articles just reporting qualitative considerations. These findings are discouraging considering that solubility and/or permeability issues^11^ are the first obstacles to design new oral drugs. Moreover, molecular properties, such as lipophilicity, which are common markers of in vitro ADME in early drug discovery, are rarely considered. The paucity of studies addressing physicochemical properties, solubility, and cell penetration could be related to the evidence that PROTACs are beyond Rule of 5 (bRo5) compounds and thus methods tailored for small molecules must be still optimized in this chemical space.^12,13^ Another possible explanation could reside in the traditionally different expertise field of biological-oriented laboratories, often far from physicochemical properties measurement.

PROTAC ternary complex (TC) formation was also not extensively studied: less than 40% of the considered articles. This could be related to the still poorly understood relationship between TC formation and degradation success rate.^14^ With the necessary biophysical measurements often being costly and requiring specialized expertise, it is tempting to hypothesize that the study of TC formation is not considered as necessary in current studies. However, a TC needs to be formed to have POI degradation; thus, this aspect should be addressed too.

Finally, as expected, most of the papers reported both ubiquitination and degradation data: 90% and 100%, respectively.

In this particular scenario, the paper obtained from *ACS Med. Chem. Lett.* presented a similar portrayal to that of *J. Med. Chem.* (Figure S2; Tables S3, S4). Physicochemical descriptors were computed in 30% of the papers, but no experimental determination is described. Solubility was never reported, and cell penetration was assessed only by 20% of the publications. Both journals exhibited a similar frequency in measuring TC formation and degradation profiles. Notably, ubiquitination was less assessed in the *J. Med. Chem.Lett.* than in *J. Med. Chem.*

## Implemented Assays

**Figure 2:**
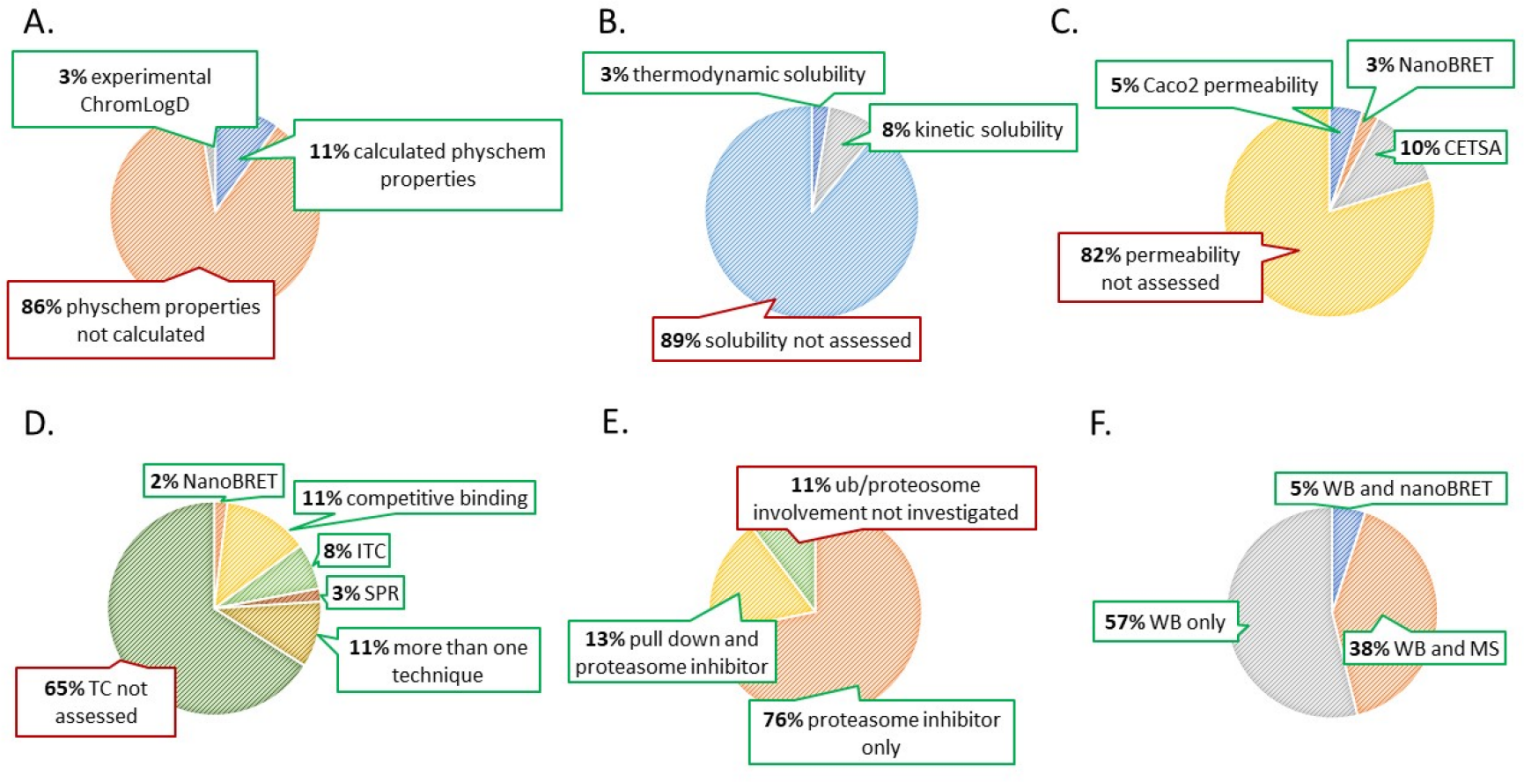
Pie charts showing the experimental approaches used to measure (A) physicochemical properties, (B) kinetic and thermodynamic solubility, (C) cell penetration, (D) ternary complex formation, (E) involvement of the ubiquitin-proteasome system, and (F) degradation.

Infographics (Figure 2) was used to provide an overview of the experimental techniques adopted in the 37 papers and referring to steps 0–5 (Figure 1).

Physicochemical descriptors mostly consist of lipophilicity data of computational nature apart from one publication where lipophilicity was experimentally assessed through the ChromLogD method (Figure 2A). A few articles employed SwissADME to predict the topological polar surface area (TPSA), the number of H-bond acceptors and donors (HBA, HBD), the molecular weight (MW) and flexibility-related descriptors (e.g., the number of rotatable bonds, nRotB).

Solubility (Figure 2B) was primarily measured through kinetic assays (8%), although one publication evaluated thermodynamic solubility.

Cell penetration was assessed through Caco-2 cell lines in 5% of cases, 10% performed CEllular Thermal Shift Assay (CETSA), and the remaining 3% employed NanoBRET (Figure 2C). Cell-based models and parallel artificial membrane permeability assay (PAMPA) are the most used assays to measure permeability in drug discovery programs. However, for bRo5 molecules and specifically for PROTACs, no correlation between PAMPA and Caco2 is found, as reported by Wittwer.^13^ This could be related to the fact that PAMPA systems only provide permeability values due to passive permeation mechanisms whereas cell-based models, including Caco2, MDCKII, or LLC-PK1, allow to also estimate the active transport contribution. Probably for this reason, PAMPA measurements are not retrieved in the 37 considered papers. CETSA is an indirect method to prove PROTAC-target engagement at a cellular level.^15^ After incubation with the degrader, a temperature gradient is applied causing protein denaturation. The degrader-protein interaction stabilizes the target protein creating a shift in the target melting curve; while the unbound protein precipitates, the one stabilized by the ligand interaction remains in solution and can be quantified after cell lysis. NanoBRET is a quite recent proprietary technology that combines CRISPR-Cas9 endogenous tagging with bioluminescence resonance energy transfer.^16^ It allows kinetic real-time measurements. This technology relies on the creation of a bioluminescent fusion protein (i.e., the target) that can compromise the localization, structure, and function of the native protein. For this reason, each target requires a specific validation process that could discourage NanoBRET application. Notably, poor solubility might affect all cell penetration assays, but a combined solubility/permeability analysis was never described in the retrieved papers.

Ternary complex formation has been proven to have a considerable impact on target degradation efficacy and efficiency.^17,18^ Many biochemical and biophysical assays can be performed to characterize the ternary complex in terms of cooperativity, stability, binding affinities, and kinetics of formation: 11% of the related papers employed competitive assays, 8% isothermal titration calorimetry (ITC), and 3% surface plasmon resonance (SPR) (Figure 2D). The former is a calorimetry-based technique measuring heat variations following the ligand–protein interaction. This label-free technique allows to study the ternary complex stability and cooperativity in solution in a direct way. SPR is a spectroscopic indirect method that requires the immobilization of the target on the chip surface to assess and quantify the binding. Indeed, the interaction between the molecules in solution and the protein immobilized on the sensor chip causes a change in the refractive index of the medium and the intensity of the reflected light, allowing to study the binding kinetics in real-time.^19^ Both ITC and SPR require highly specific instrumentation and substantial expertise in the field.^19^ Interestingly, more than 10% of the 37 papers combined two or more techniques to validate TC formation (including AlphaLISA bead-based immunoassay as well). This procedure is highly recommended and the combination of direct with indirect methods can also provide a significative gain in knowledge.

All works investigated target degradation (Figure 2F) via Western Blotting (WB), which can be considered the current gold standard for PROTAC degradation assessment. Alongside WB, 5% of the papers opted for NanoBRET (see above), and 38% performed a mass spectrometry (MS) analysis to quantify the target and identify degraders off targets (the KinomeScan technique was also used to evaluate degraders kinase selectivity). The large use of WB is understandable: this assay allows one to contain the costs and timing, and the major pitfalls are related to the difficulty in finding adequate antibodies. However, WB is far from being flawless; It is a low throughput assay providing semiquantitative results. It is a lytic end-point assay, and thus, it cannot be performed on live cells. Notably a default WB protocol to assess protein degradation is not available yet. The incubation time is quite heterogeneous; however, 24 h of incubation seems to be the preferred setup (more than 60% of all articles); and just 40% opted for a smaller (6, 8, or 16 h) or a longer (48 or 72 h) amount of time (data not shown). The number of cell lines used to test the target degradation also greatly varies and mainly depends on the author’s purpose and availability. One half of the studies tested degrader efficacy on more than two cell lines, while the other half showed the PROTAC efficacy in just one cell line. Overall, the definition of a default degradation protocol by an expert consortium is highly recommended for the next future.

To validate and confirm the mechanism of action of PROTACs it is necessary to demonstrate the actual involvement of the ubiquitin-proteasome system (Figure 2E). Only a couple of studies did not verify it; on the contrary, the vast majority preincubated the cells with MG132, a proteasome inhibitor, or MLN-4924, a NEDD8 activating enzyme inhibitor, to rescue target protein level in the presence of the degrader. Co-immunoprecipitation or pull-down assay were performed to verify target ubiquitination as well.

Once again, regarding the utilized assays, *ACS Med Chem Lett.* and *J. Med. Chem.* offer analogous information. Physicochemical descriptors were calculated with various tools (Figure S3) but never measured. Also the Letters assessed permeability and target engagement mainly via the Caco-2 cell system and CETSA, respectively. Ternary complex formation was poorly assessed: one publication applied competitive binding, one implemented AlphaLISA, and another employed both techniques. Also target protein degradation shows a similar overall trend in the two journals: 60% performed WB only, three Letters investigated target degradation using the NanoBRET assay, and only one used MS. Ubiquitination was mainly assessed by proteosome inhibition.

## Take Home Message

Our analysis highlights the absence of a default PROTAC experimental pipeline and emphasizes the need for collective efforts in establishing one. More physicochemical, solubility, and permeability data should be produced. We are aware of the experimental challenges associated with the assessment of in vitro ADME for bRo5, but efforts should be made along these lines to optimize the synthetic effort. Since TC formation strongly impacts degrader efficacy, we also encourage the measurement of TC formation in early drug discovery for at least a pool of representative PROTACs of the investigated series. Data quality is essential in any step of the PROTAC pipeline. Whenever feasible, a consensus approach is appreciated for evaluating a specific attribute, even though it may require additional allocation of resources. Data arising from different pipeline steps are often considered separately and not in combination, e.g., solubility measurements and degradation data; we strongly encourage researchers to consider all these aspects as different sides of the same coin. Finally, ineffective degraders are as relevant as highly potent ones; if the entire experimental pipeline is followed and properly documented, we can learn from our failures, since they can show us what needs to be improved. Moreover, this could help avoid blind starts with new targets. This is particularly true in the infancy of a given research field, like PROTACs are.

## Supporting Information Available

Main content of the *J. Med. Chem.* papers; publication date and DOIs of the *J. Med. Chem.* papers analyzed; main content of the A*CS Med. Chem. Lett.*; publication date and DOI of the *ACS Med. Chem. Lett.*; temporal distribution of the *J. Med. Chem.* publications, target distribution of the pertinent articles, target classified according to their biological function, in vitro and in vivo proof of concept experiments; temporal distribution of *ACS Med. Chem. Lett.* publications, target distribution of the pertinent publications, target classified according to their biological function, main steps, and relative frequencies overall the pipeline; pie charts schematizing the experimental approaches used to measure physicochemical properties, permeability, ternary complex formation, involvement of the ubiquitin-proteasome system, and degradation (PDF)

## Abbreviations

PROTACs: proteolytic targeting chimeras
POI: protein of interest
bRo5: beyond Rule of 5
TC: ternary complex
TPSA: topological polar surface area
HBA, HBD: H-bond acceptors and donors
nRotB: number of rotatable bonds
CETSA: performed CEllular Thermal Shift Assay
PAMPA: parallel artificial membrane permeability assay
ITC: isothermal titration calorimetry
SPR: surface plasmon resonance
WB: Western blotting
MS: mass spectrometry
