# Supplementary material for: The Quest for Oral PROTAC drugs: Evaluating the Weaknesses of the Screening Pipeline
Source: ACS Med Chem Lett. 2023 Jul 3;14(7):879–83. doi: 10.1021/acsmedchemlett.3c00231 (PMC10351046; doi:10.1021/acsmedchemlett.3c00231)

The quest for oral PROTAC drugs: evaluating the weaknesses of the screening pipeline

Supporting Material

Giulia Apprato, Giuseppe Ermondi and Giulia Caron*

Molecular Biotechnology and Health Sciences Dept., University of Torino, Piazza Nizza, 44, 10126 Torino, Italy
*Corresponding author: *giulia.caron@unito.it*

Table of contents:

Bibliographic research strategy.
Table S1. Main content of the 37 papers of Journal of Medicinal Chemistry (DOIs in Table S2)
Table S2. Publication date and DOIs of the 37 Journal of Medicinal Chemistry papers.
Table S3. Main content of the 10 papers of the ACS Medicinal Chemistry Letters.
Table S4. Publication date and DOIs of the 10 ACS Medicinal Chemistry Letters publications.
Figure S1. A) Temporal distribution of the Journal of Medicinal Chemistry publications (January 2021-March 2023), including articles and perspectives not identified as pertinent for the analysis; B) target distribution of pertinent articles only; C) Target classified according to their biological function; D) in vitro and in vivo proof of concept experiments.
Figure S2. A) Temporal distribution of the ACS Medicinal Chemistry Letters publications (January 2021-March 2023), including articles and perspectives not identified as pertinent for the analysis; B) target distribution of pertinent publications only; C) Target classified according to their biological function; D) Main steps and their relative frequency as calculated from data reported in the retrieved papers.
Figure S3. Pie charts schematizing the experimental approaches used to measure A) physicochemical properties, B) permeability, C) ternary complex formation, D) involvement of the ubiquitin-proteasome system and E) degradation.

Bibliographic research strategy

The bibliographic research was performed on March 15th 2023; “PROTAC” or “degrader” were used as key words in Journal of Medicinal Chemistry research. All the publications published between January 2021 and March 2023 were considered (112 journal articles were retrieved). An accurate selection of the pertinent publications was done. Articles reporting PROTACs data were considered as pertinent. 37 pertinent publications were retrieved, and their content is summarized in Table S1.

A second bibliographic research was conducted on 1st of June 2023 in ACS Medicinal Chemistry Letters using the same key words, considering the same time interval and criteria of pertinence to identify relevant publications. Overall, 59 publications were retrieved, including 23 patent highlights, 17 letters, 6 In this Issue, 4 microperspectives, 3 innovations, 2 viewpoints, 1 editorial, 1 featured letter, 1 note and 1 technology note. However, only 10 publications were identified as pertinent (9 letters and the technology note), while the patent highlights were not taken into account.

Table S1. Main content of the 37 Journal of Medicinal Chemistry papers (DOIs in Table S2).

Ref.	Target	E3 ligase	Physchem	Solubility	Target engagement		Target degradation			Ternary complex formation				Ub/proteasome		Cytotoxicity assay	In vivo	
					Caco2/ BRET	CETSA	WB	nanoBRET	MS	nanoBRET	AlphaLISA	Competitive assay	ITC/ SPR	pull-down/ co-IP	Prot. Inh.		PK	xenograft
**1**	SOS1	VHL	no	no	no	no	yes	no	no	no	no	yes	SPR	no	yes	yes	yes	yes
**2**	hCAII	CRBN	no	no	no	no	yes	no	no	no	no	no	no	no	yes	no	no	no
**3**	AXL	BOTH	no	no	no	no	yes	no	no	no	no	no	no	yes	yes	yes	no	yes
**4**	NCL	CRBN	no	no	no	no	yes	no	no	no	no	no	no	yes	yes	yes	no	no
**5**	NAMPT	VHL	no	no	no	yes	yes	no	yes	no	no	no	no	no	yes	yes	yes	yes
**6**	LDH	VHL	no	no	no	no	yes	no	yes	no	no	no	no	no	yes	yes	yes	no
**7**	HDAC8	BOTH	no	no	no	no	yes	no	yes	no	no	no	no	no	yes	yes	no	yes
**8**	HDAC6	CRBN	yes	no	no	no	yes	yes	yes	no	no	no	no	no	yes	yes	no	no
**9**	SOS1	CRBN	no	no	no	no	yes	no	yes	no	no	no	no	no	yes	yes	no	no
**10**	NAMPT	VHL	no	no	no	yes	yes	no	no	no	no	yes	no	no	yes	yes	yes	yes
**11**	PAK1	CRBN	no	no	no	no	yes	no	yes	no	no	no	no	no	yes	yes	no	no
**12**	IDO1	BOTH	no	no	no	no	yes	no	no	no	no	no	ITC	yes	yes	no	yes	yes
**13**	BRD4	BOTH	no	yes(t)	no	no	yes	no	no	no	no	yes	no	no	no	no	no	no
**14**	AKT	BOTH	no	no	no	no	yes	no	yes	no	no	no	no	no	yes	yes	yes	yes
**15**	HDAC4	VHL	no	yes(k)	yes	no	yes	no	no	no	no	no	no	no	yes	yes	no	no
**16**	CDK12/13	CRBN	no	yes(k)	no	no	yes	no	yes	no	no	no	no	no	yes	yes	yes	yes
**17**	NSD2	CRBN	no	no	no	no	yes	no	no	no	no	no	ITC	no	yes	yes	yes	no
**18**	EGFR	BOTH	no	no	no	no	yes	no	no	no	no	no	no	yes	yes	yes	no	no
**19**	BTK	CRBN	yes	no	no	no	yes	no	yes	no	no	no	no	no	yes	yes	yes	yes
**20**	HA	BOTH	no	no	no	no	yes	no	no	no	no	no	SPR	no	yes	yes	yes	yes
**21**	STING	CRBN	yes	no	no	no	yes	no	no	no	no	no	no	no	no	yes	yes	yes

**22**	HDAC1/2/3	VHL	yes	no	no	no	yes	no	no	no	no	no	no	no	yes	yes	no	no
**23**	SOS1	VHL	no	yes(k)	no	no	yes	no	no	no	no	yes	SPR	no	yes	yes	yes	no
**24**	AKT	BOTH	no	no	no	no	yes	no	no	no	no	no	ITC	no	yes	yes	yes	yes
**25**	TTK	BOTH	no	no	no	no	yes	no	no	no	no	no	no	no	yes	yes	yes	yes
**26**	AKT	BOTH	no	no	no	no	yes	no	yes	no	no	no	no	no	yes	yes	yes	no
**27**	FOXM1	CRBN	no	no	no	no	yes	no	no	no	no	no	no	yes	yes	yes	yes	yes
**28**	CDK9	CRBN	no	no	no	no	yes	no	no	no	no	no	no	no	yes	yes	yes	yes
**29**	BCL-XL	VHL	no	no	no	yes	yes	no	no	yes	yes	no	no	no	yes	yes	no	no
**30**	AR	CRBN	no	no	no	no	yes	no	no	no	no	no	no	no	yes	yes	yes	yes
**31**	RIPK2	IAP	no	no	no	no	yes	no	no	no	no	yes	no	no	no	no	yes	no
**32**	IRAK1	VHL	no	no	no	no	yes	no	yes	no	no	yes	no	no	yes	yes	no	no
**33**	EZH2	BOTH	no	no	yes	no	yes	no	no	no	no	no	no	no	yes	yes	no	yes
**34**	ALK	CRBN	no	no	no	no	yes	no	no	no	no	no	no	no	no	yes	yes	yes
**35**	PAN-kinase	CRBN	no	no	no	no	yes	no	yes	no	no	no	no	no	yes	yes	yes	no
**36**	WDR5	BOTH; MDM2	no	no	yes	no	yes	yes	yes	yes	no	no	ITC	no	yes	yes	no	no
**37**	STAT3	CRBN	no	no	no	yes	yes	no	yes	no	no	no	no	no	yes	yes	no	no

Table S2. Publication date and DOI of the 37 Journal of Medicinal Chemistry papers.

Ref.	Publication date	DOI
**1**	March 10, 2023	10.1021/acs.jmedchem.3c00075
**2**	February 3, 2023	10.1021/acs.jmedchem.2c01843
**3**	January 25, 2023	10.1021/acs.jmedchem.2c01682
**4**	January 6, 2023	10.1021/acs.jmedchem.2c01557
**5**	December 23, 2022	10.1021/acs.jmedchem.2c01990
**6**	December 20, 2022	10.1021/acs.jmedchem.2c01505
**7**	December 14, 2022	10.1021/acs.jmedchem.2c00739
**8**	December 6, 2022	10.1021/acs.jmedchem.2c01659
**9**	December 2, 2022	10.1021/acs.jmedchem.2c01300
**10**	November 28, 2022	10.1021/acs.jmedchem.2c01243
**11**	November 23, 2022	10.1021/acs.jmedchem.2c00756
**12**	November 21, 2022	10.1021/acs.jmedchem.2c00771
**13**	November 2, 2022	10.1021/acs.jmedchem.2c01218
**14**	October 5, 2022	10.1021/acs.jmedchem.2c01454
**15**	September 13, 2022	10.1021/acs.jmedchem.2c01149
**16**	August 8, 2022	10.1021/acs.jmedchem.2c00384
**17**	July 27, 2022	10.1021/acs.jmedchem.2c00807
**18**	June 8, 2022	10.1021/acs.jmedchem.2c00345
**19**	June 7, 2022	10.1021/acs.jmedchem.2c00324
**20**	May 17, 2022	10.1021/acs.jmedchem.1c02013
**21**	April 22, 2022	10.1021/acs.jmedchem.1c01948
**22**	March 16, 2022	10.1021/acs.jmedchem.1c02179
**23**	March 1, 2022	10.1021/acs.jmedchem.1c01774
**24**	February 4, 2022	10.1021/acs.jmedchem.1c02165
**25**	January 27, 2022	10.1021/acs.jmedchem.1c01768
**26**	December 2, 2021	10.1021/acs.jmedchem.1c01476
**27**	November 23, 2021	10.1021/acs.jmedchem.1c01069
**28**	September 20, 2021	10.1021/acs.jmedchem.1c01350
**29**	September 17, 2021	10.1021/acs.jmedchem.1c00517
**30**	September 2, 2021	10.1021/acs.jmedchem.1c00900
**31**	August 25, 2021	10.1021/acs.jmedchem.1c01118
**32**	July 19, 2021	10.1021/acs.jmedchem.1c00103
**33**	July 1, 2021	10.1021/acs.jmedchem.1c00460
**34**	June 28, 2021	10.1021/acs.jmedchem.1c00270
**35**	June 17, 2021	10.1021/acs.jmedchem.1c00373
**36**	May 13, 2021	10.1021/acs.jmedchem.1c00146
**37**	January 28, 2021	10.1021/acs.jmedchem.0c01897

Table S3. Main content of the 10 ACS Medicinal Chemistry Letters publications (DOIs in Table S4).

Ref	Target	E3 ligase	Physchem	Solubility	Target engagement		Target degradation			Ternary complex formation				Ub/proteasome		Cytotoxicity assay	In vivo	
					Caco-2	CETSA	WB	NanoBRET	MS	NanoBRET	AlphaLISA	Comp. assay	ITC SPR	Pulldown Co-IP	Prot. Inhib.		PK	Xenograft
1	LCK	CRBN	no	no	yes	no	yes	yes	no	no	yes	no	no	no	no	yes	no	no
2	MEK1/2	both	no	no	no	no	yes	no	no	no	no	no	no	no	yes	yes	no	no
3	FLT3	VHL, CRBN, IAP	no	no	no	no	yes	no	no	no	no	no	no	no	yes	yes	no	no
4	BRD4	CRBN	no	no	no	yes	yes	yes	no	yes	yes	yes	ITC	no	yes	yes	no	no
5	BRD4	CRBN	yes	no	no	no	no	yes	no	no	no	no	no	no	no	no	no	no
6	JAK2/3	CRBN	no	no	no	no	yes	no	no	no	no	yes	no	no	no	yes	no	yes
7	EGFR	VHL	no	no	no	no	yes	no	no	no	no	no	no	no	yes	yes	no	no
8	CDK4/6	CRBN	yes	no	no	no	yes	no	no	no	no	no	no	no	no	no	no	no
9	STAT3	CRBN	yes	no	no	no	yes	no	yes	no	no	no	no	no	no	yes	yes	yes
10	HPGDS	CRBN, IAP	no	no	no	no	yes	no	no	no	no	no	no	no	yes	yes	no	no

Table S4. Publication date and DOI of the 10 ACS Medicinal Chemistry Letters publications.

Ref	Publication date	DOI
**1**	January 4, 2023	10.1021/acsmedchemlett.2c00436
**2**	December 8, 2022	10.1021/acsmedchemlett.2c00446
**3**	November 4, 2022	10.1021/acsmedchemlett.2c00402
**4**	September 29, 2022	10.1021/acsmedchemlett.2c00300
**5**	June 20, 2022	10.1021/acsmedchemlett.2c00124
**6**	February 21, 2022	10.1021/acsmedchemlett.1c00650
**7**	January 14, 2022	10.1021/acsmedchemlett.1c00645
**8**	October 18, 2021	10.1021/acsmedchemlett.1c00368
**9**	May 10, 2021	10.1021/acsmedchemlett.1c00155
**10**	January 14, 2021	10.1021/acsmedchemlett.0c00605

Figure S1. A) Temporal distribution of the Journal of Medicinal Chemistry publications (January 2021-March 2023), including articles and perspectives not identified as pertinent for the analysis; B) Target distribution of pertinent articles only; C) Target classified according to their biological function; D) *In vitro* and *in vivo* proof of concept experiments.

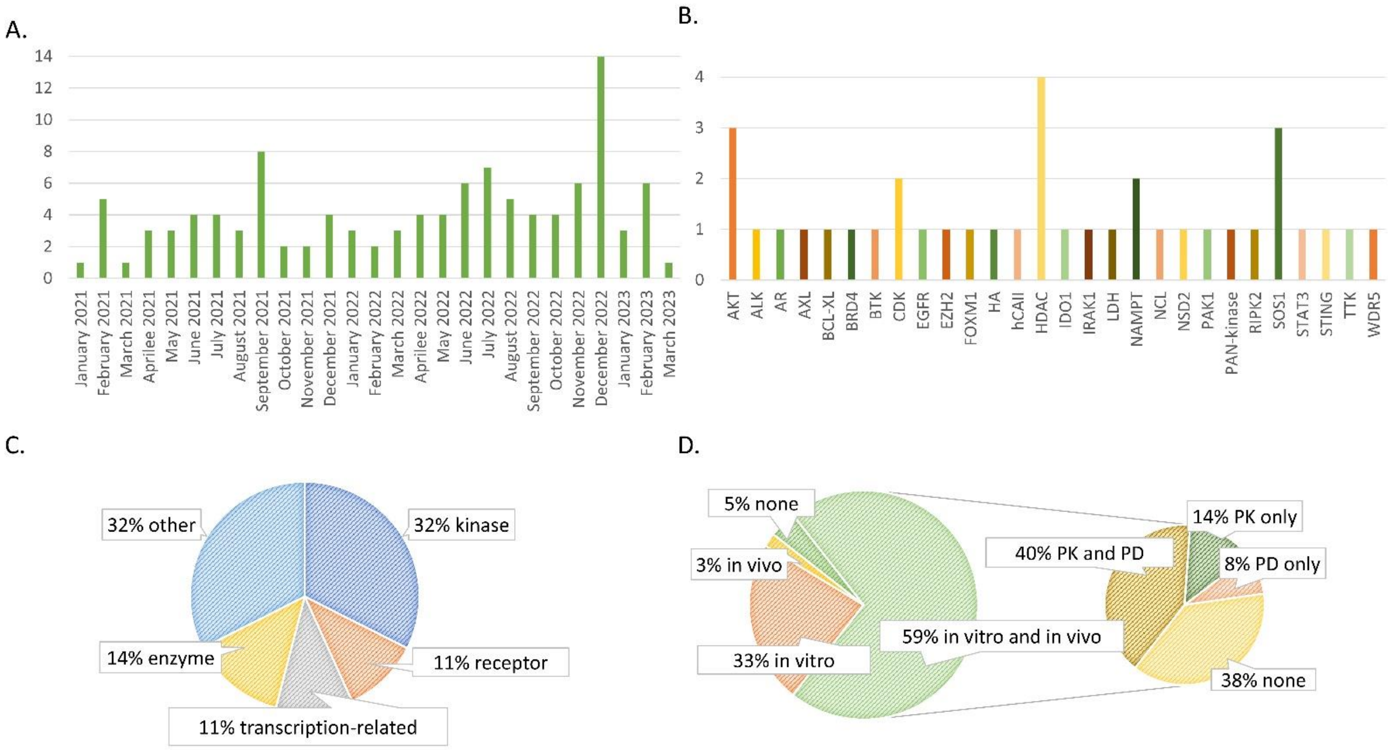

Figure S2. A) Temporal distribution of the ACS Medicinal Chemistry Letters publications (January 2021-March 2023), including publications not identified as pertinent for the analysis; B) Target distribution of pertinent publications only; C) Target classified according to their biological function; D) Main steps and their relative frequency as calculated from data reported in the retrieved papers.

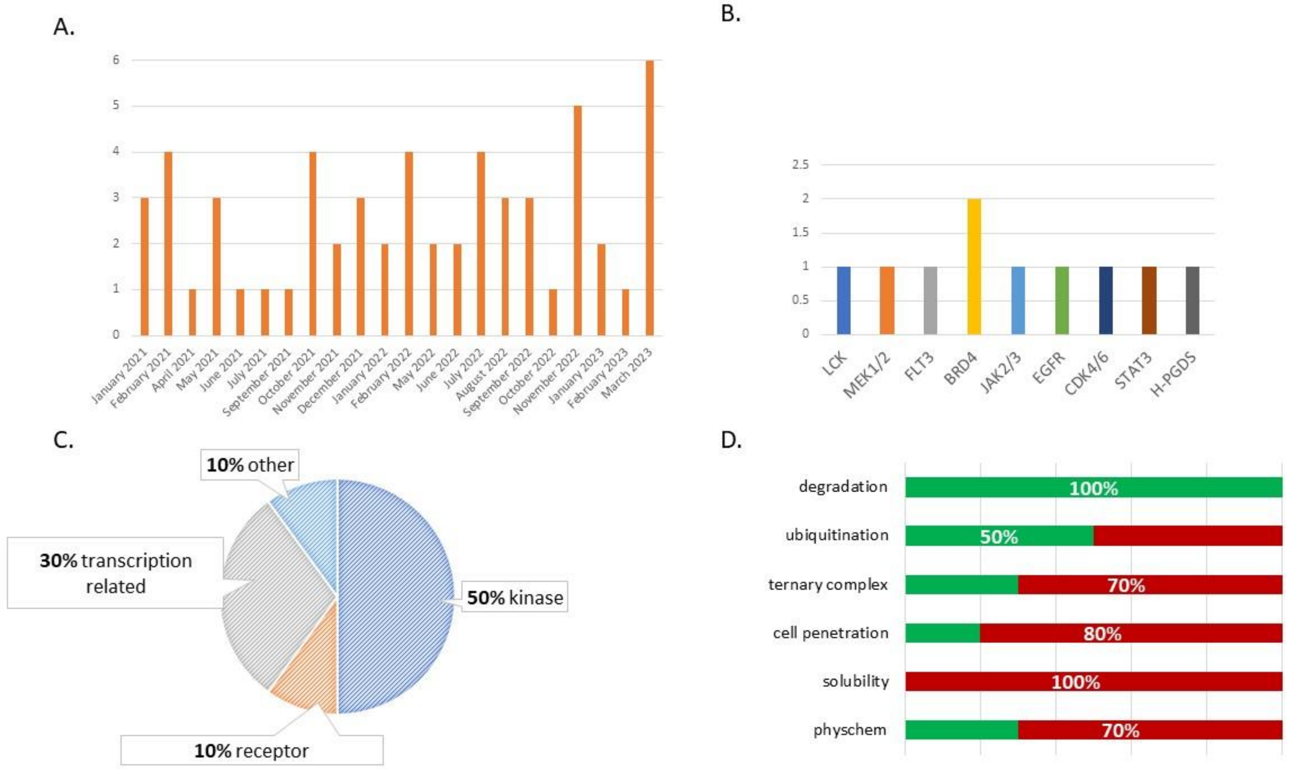

Figure S3. Pie charts schematizing the experimental approaches used to measure A) physicochemical properties, B) permeability, C) ternary complex formation, D) involvement of the ubiquitin-proteasome system and E) degradation.

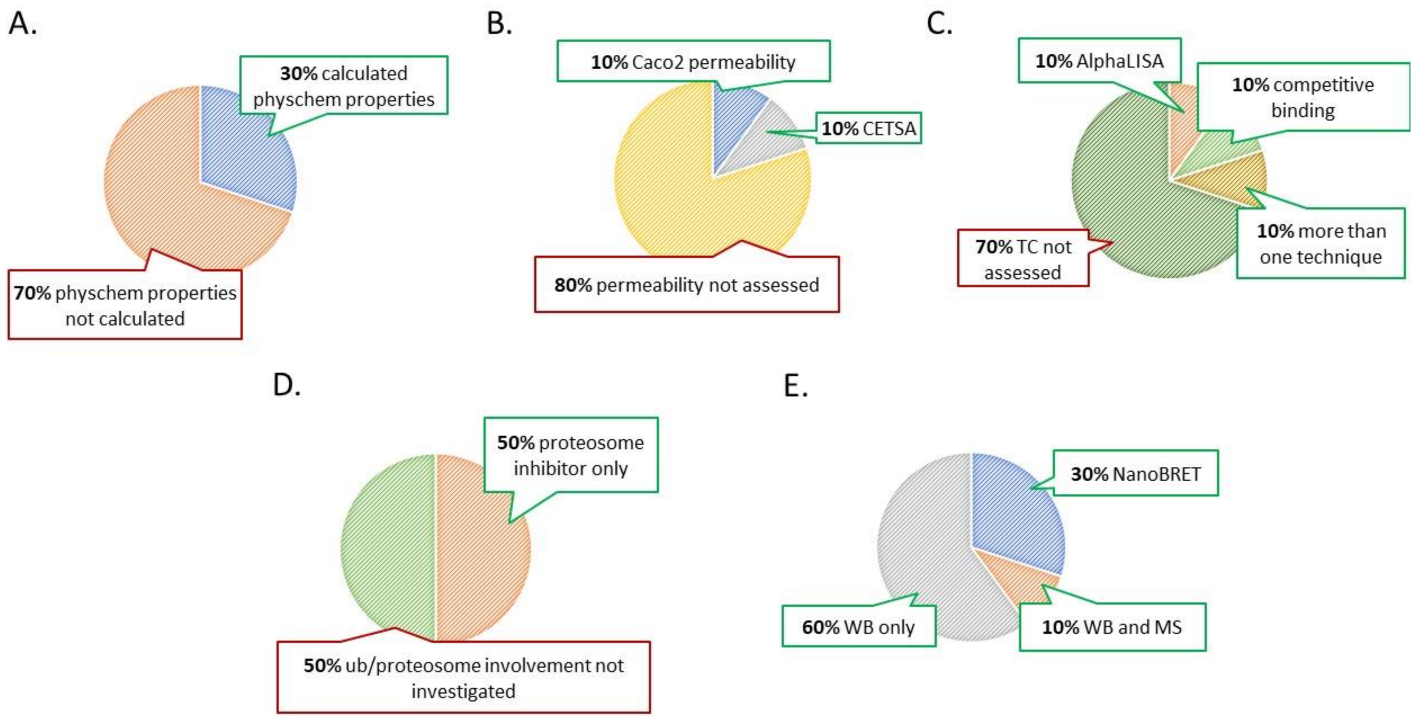

Supplement: Supplementary file 1 — ml3c00231_si_001.pdf [file ml3c00231_si_001.pdf]
